# Hepatoprotective Effect of Polysaccharides Isolated from *Dendrobium officinale* against Acetaminophen-Induced Liver Injury in Mice via Regulation of the Nrf2-Keap1 Signaling Pathway

**DOI:** 10.1155/2018/6962439

**Published:** 2018-07-18

**Authors:** Guosheng Lin, Dandan Luo, Jingjing Liu, Xiaoli Wu, Jinfen Chen, Qionghui Huang, Lingye Su, Lei Zeng, Hongfeng Wang, Ziren Su

**Affiliations:** ^1^Mathematical Engineering Academy of Chinese Medicine, Guangzhou University of Chinese Medicine, Guangzhou 510006, China; ^2^Guangdong Provincial Key Laboratory of New Drug Development and Research of Chinese Medicine, Guangzhou University of Chinese Medicine, Guangzhou 510006, China; ^3^Postdoctoral Programme, Department of Second Institute of Clinical Medicine, Guangzhou University of Chinese Medicine, Guangzhou 510006, China; ^4^Guangdong Provincial Key Laboratory of Silviculture, Protection and Utilization, Guangdong Academy of Forestry, Guangzhou 510520, China

## Abstract

The effect of polysaccharides isolated from *Dendrobium officinale* (DOP) on acetaminophen- (APAP-) induced hepatotoxicity and the underlying mechanisms involved are investigated. Male Institute of Cancer Research (ICR) mice were randomly assigned to six groups: (1) control, (2) vehicle (APAP, 230 mg/kg), (3) *N*-acetylcysteine (100 mg/kg), (4) 50 mg/kg DOP, (5) 100 mg/kg DOP, and (6) 200 mg/kg DOP. Alanine aminotransferase (ALT) and aspartate aminotransferase (AST) levels in the serum and glutathione (GSH), malondialdehyde (MDA), catalase (CAT), total antioxidant capacity (T-AOC), myeloperoxidase (MPO), and reactive oxygen species (ROS) levels in the liver were determined after the death of the mice. The histological examination of the liver was also performed. The effect of DOP on the Kelch-like ECH-associated protein 1- (Keap1-) nuclear factor erythroid 2-related factor 2 (Nrf2) signaling pathway was evaluated using Western blot analysis and real-time polymerase chain reaction (PCR). The results showed that DOP treatment significantly alleviated the hepatic injury. The decrease in ALT and AST levels in the serum and ROS, MDA, and MPO contents in the liver, as well as the increases in GSH, CAT, and T-AOC in the liver, were observed after DOP treatment. DOP treatment significantly induced the dissociation of Nrf2 from the Nrf2−Keap1 complex and promoted the Nrf2 nuclear translocation. Subsequently, DOP-mediated Nrf2 activation triggered the transcription and expressions of the glutamate–cysteine ligase catalytic (GCLC) subunit, glutamate–cysteine ligase regulatory subunit (GCLM), heme oxygenase-1 (HO-1), and NAD(P)H dehydrogenase quinone 1 (NQO1) in APAP-treated mice. The present study revealed that DOP treatment exerted potentially hepatoprotective effects against APAP-induced liver injury. Further investigation about mechanisms indicated that DOP exerted the hepatoprotective effect by suppressing the oxidative stress and activating the Nrf2−Keap1 signaling pathway.

## 1. Introduction

Drug-induced liver injury (DILI) has become one of the most frequent causes leading to cessation of drug test in clinical trials, restrictions on drug use, and withdrawal of approved drugs [1]. The overall occurrence of DILI is between 0.00001% and 0.01% patient-years [2, 3]. DILI can broadly be divided into predictable and dose-dependent, such as acetaminophen (paracetamol, APAP), and unpredictable or idiosyncratic DILI (IDILI). IDILI is a significant health problem due to its unpredictability, potential cause for mortality, and poorly understood pathogenesis [4, 5]. APAP overdose accounts for 50% of the acute liver failure cases in a predictable, dose-dependent, and intrinsically hepatotoxic manner [6, 7]. Moreover, nearly half of the liver transplantation cases caused by DILI in the United States are attributed to APAP alone or in combination with other drugs [8]. APAP is an over-the-counter drug widely used as antipyretic and analgesic [9]. APAP is safe at therapeutic doses, but it is easy to be overused due to the individual difference. Furthermore, liver injury induced by APAP overdose occurs quickly within 24–48 h after ingestion [10]. At present, the clinical treatment for APAP-induced hepatotoxicity is extremely limited. *N*-Acetylcysteine (NAC) is the most effective first-line antidote [11], but its effectiveness is restricted to the early stages of APAP-induced toxicity [12]. Hence, more effective and safe drugs to relieve the liver injury induced by APAP need to be urgently developed.

Recently, herbal products have attracted great attention as a major part of alternative medicine [13, 14]. A large population chooses naturally derived medicines to maintain health and treat diseases [15]. Companies are also making attempts to discover new drugs of plant origin and perform structural modifications. Herbal drugs promote the regeneration of liver cells and accelerate the healing process in liver disorders [16].

*Dendrobium officinale* Kimura et Migo, called “Tie-Pi-Shi-Hu” in Chinese, is an original food material and medicinal plant. As a traditional Chinese medicine in China, *D. officinale* is generally used to reinforce stomach function and generate body fluid [17]. Modern pharmacological studies indicate that *D. officinale* displays immunomodulatory, antidiabetic, anti-inflammatory, anticancer, hepatoprotective, and antioxidative activities [18, 19]. Chemical composition studies reveal that *D. officinale* mainly consists of polysaccharides, alkaloids, amino acids, and trace elements [18, 19]. Among these chemical ingredients, polysaccharides isolated from *D. officinale* have an active role in food and health in human diet [20]. In particular, polysaccharides extracted from *D. huoshanense*, another species of *Dendrobium*, displayed hepatoprotective activities against ethanol- and carbon tetrachloride-induced liver injuries [21, 22]. However, the effect and the mechanism of action of polysaccharides isolated from *D. officinale* (DOP) against APAP-induced liver injury remain unknown.

The present study is aimed at investigating the hepatoprotective effects of DOP against APAP-induced liver injury in mice. Specifically, the levels of alanine aminotransferase (ALT) and aspartate aminotransferase (AST) in serum were measured to assess the liver injury. Glutathione (GSH), malondialdehyde (MDA), reactive oxygen species (ROS), and myeloperoxidase (MPO) levels in the liver were determined to demonstrate oxidative damage. Catalase (CAT) and total antioxidant capacity (T-AOC) were also examined to test the antioxidative capacity. Also, the underlying mechanisms of the Nrf2–Keap1 signaling pathway were investigated using Western blot analysis and real-time polymerase chain reaction (PCR).

## 2. Materials and Methods

### 2.1. Chemicals and Reagents

*D. officinale* was kindly supplied by the Guangdong Academy of Forestry (Guangzhou, China). APAP was purchased from Shanghai Macklin Biochemical Co., Ltd. (Shanghai, China). NAC was obtained from Guangzhou Feibo Biological Technology Co., Ltd. (Guangzhou, China). Biochemical assay kits of ALT (C009-2), AST (C0010-2), GSH (A006-1), MDA (A003-1), MPO (A004), CAT (A007-1), and T-AOC (A015) were purchased from Nanjing Jiancheng Bioengineering Institute (Nanjing, China). The mouse tissue ROS enzyme-linked immunosorbent assay (ELISA) kit (E-20634) was bought from Beijing Cheng Lin Biological Technology Co., Ltd. (Beijing, China). Nrf2 (sc-365949) and Keap1 (sc-365626) antibodies were obtained from Santa Cruz Biotechnology Inc. (CA, USA). Histone H3 (E021130-03), *β*-actin (E021020-03), and secondary antibodies (E030110-01) were provided by EarthOx, LLC (CA, USA). Primers of glutamate–cysteine ligase catalytic subunit (GCLC), glutamate–cysteine ligase regulatory subunit (GCLM), heme oxygenase-1 (HO-1), NAD(P)H dehydrogenase quinone 1 (NQO1), and glyceraldehyde-3-phosphate dehydrogenase (GAPDH) were provided by Shanghai Sangon Biotech Co., Ltd. (Shanghai, China). TRIzol Reagent was purchased from Invitrogen (CA, USA). ChamQ SYBR qPCR Master Mix was provided by Vazyme Biotech Co., Ltd. (Nanjing, China). Except as otherwise expressly stated, other chemicals and reagents were used in analytical grade for meeting the needs of the study.

### 2.2. Preparation of DOP from *D. officinale*

Polysaccharides isolated from *D. officinale* (DOP) were prepared as previously described [23, 24]. First, *D. officinale* (100 g) was crushed and extracted thrice with distilled water (1 : 20, wt/wt) for 2 h. The combined extracts were filtered to remove impurities. Then, the supernatant was concentrated and deproteinized by adding the Sevag reagent. The alcohol concentration of the obtained liquid was adjusted to be 80% using edible alcohol. Afterward, the liquid was precipitated at 4°C overnight. The precipitate was collected by centrifuging at 3000 rpm for 10 min at 4°C and redissolved in appropriate amounts of distilled water. Finally, the dissolved liquid was lyophilized using the vacuum freeze dryer (FreeZone Labconco, MO, USA) to obtain the DOP dry powder. The yield of polysaccharides in DOP was calculated as follows: polysaccharide yield = (polysaccharide weight (g)/*Dendrobium* *officinale* weight (g)) × 100%.

The total amount of soluble carbohydrates in DOP was measured by the anthrone−sulfuric acid method [25]. First, 2 mL of DOP solution (0.04 mg/mL) was mixed with 6 mL of sulfuric anthrone (1 mg/mL) and heated at 90°C for 15 min followed by cooling to room temperature. The absorbance of the reaction liquid was measured using Multiskan Spectrum (Thermo, Finland) at 625 nm. Different concentrations of D-glucose (2, 4, 6, 8, 10, and 12 mg/100 mL) were prepared to draw the standard curve according to the aforementioned method.

### 2.3. Determination of the Molecular Weight of DOP with High-Performance Gel Permeation Chromatography

The molecular weights (MWs) of DOP were evaluated by high-performance gel permeation chromatography (HPGPC) according to the literature [26]. The HPGPC system was equipped with a 1525 HPLC instrument (Waters, MA, USA), a 2414 refractive index detector (Waters), and a 717 plus automatic sampler (Waters), which were acted on TSK G-5000_PWXL_ (7.8 × 300 mm^2^, Tosoh Co., Ltd, Japan) and TSK G-3000_PWXL_ (7.8 × 300 mm^2^, Tosoh Co., Ltd) columns in series. Then, 5 mg of samples was dissolved in 2 mL of 0.02 M potassium dihydrogen phosphate buffer solution and filtered through 0.45 *μ*m filters to obtain the supernatant. Further, 10 *μ*L of supernatant was injected into the HPGPC equipment for analysis. The analysis was performed with 0.02 M KH_2_PO_4_ at a flow rate of 0.6 mL/min under the column and a detector temperature of 35°C. The eight dextran standards of known MWs were used to calculate the MW (D5200, 11,600, 23,800, 48,600, 148,000, 273,000, 410,000, and 668,000).

### 2.4. Animals and Treatment

Male ICR mice (6−8 weeks old) were purchased from Guangdong Medical Laboratory Animal Center (Foshan, China) and maintained with humidity of 50 ± 10% at 23 ± 2°C for a 12 h light/dark cycle. They were given free access to food and water. All procedures were performed in accordance with the Regulations of Experimental Animal Administration issued by the Ministry of Science and Technology of the People's Republic of China. All animal experiments were approved by the Institutional Animal Care and Use Committee of Guangzhou University of Chinese Medicine.

All mice were randomly assigned to the following six groups (*n* = 6/group): (1) control, (2) vehicle (APAP, 230 mg/kg), (3) NAC (230 mg/kg APAP + 100 mg/kg NAC), (4) low dose of DOP (230 mg/kg APAP + 50 mg/kg DOP), (5) moderate dose of DOP (230 mg/kg APAP + 100 mg/kg DOP), and (6) high dose of DOP (230 mg/kg APAP + 200 mg/kg DOP). The mice from the control and vehicle groups were orally given distilled water. The other four groups were treated with corresponding doses of NAC and DOP orally for 30 days. All mice were intraperitoneally injected with APAP 4 h after the last treatment on day 30, while the control group was injected with the same volume of normal saline. The blood of mice was collected from posterior orbital venous plexus 12 h after the APAP challenge. Then, the mice were sacrificed and liver tissues were collected followed by washing with normal saline three times. The blood and the liver tissues were used for biological and histological evaluation studies.

### 2.5. Histological Analysis

Liver tissues were fixed in 4% paraformaldehyde, embedded in paraffin, sectioned at 5 *μ*m thickness, and stained with hematoxylin and eosin (H&E) following a standard protocol with minor modifications [27]. Histopathological changes in the liver were captured with an optical microscope at 200× magnification (E100, Nikon Corporation, Tokyo Japan).

### 2.6. Determination of ALT and AST Levels in Serum

The blood samples were kept at room temperature for 2 h and then centrifuged at 3000 rpm for 10 min at 4°C to obtain serum. The ALT and AST levels in the serum were quantified using commercial kits (Nanjing Jiancheng Bioengineering Institute, Nanjing, China).

### 2.7. Determination of the GSH, MDA, CAT, T-AOC, and ROS Levels and the MPO Activity in Liver Tissues

The liver tissues were thawed and homogenized in the nine folds (g/mL) of ice-cold normal saline. The homogenate was centrifuged at 2500 rpm for 10 min at 4°C to obtain the supernatant. The GSH, MDA, CAT, T-AOC, and ROS levels and MPO activity in the supernatant were measured using commercial kits (Nanjing Jiancheng Bioengineering Institute, Nanjing) and ELISA kit [28] according to the manufacturer's protocols.

### 2.8. Western Blot Analysis

Briefly, the liver tissues were homogenized using radioimmunoprecipitation assay (RIPA) buffer (R0020) (Solarbio, Beijing, China) containing phenylmethanesulfonyl fluoride (PMSF) (Beyotime, Shanghai, China). The cytosolic and nuclear proteins were extracted using a KeyGen nuclear kit and a cytoplasmic protein extraction kit (KeyGen Biotech, Jiangsu, China) according to the manufacturer's protocols, respectively. Protein concentration was quantified using a Bicinchoninic Acid Kit (BestBio, Shanghai, China). Equal amounts of protein samples were separated using sodium dodecyl sulfate–polyacrylamide gel electrophoresis and then transferred onto a polyvinylidene difluoride membrane. After being blocked in 5% skimmed milk powder in Tris-buffered saline and Tween 20 (TBST) for 1 h at room temperature, the membranes were incubated with the following primary antibodies at 4°C overnight: Keap1 (1 : 200 dilution), Nrf2 (1 : 200 dilution), Histone H3 (1 : 1000 dilution), and *β*-actin (1 : 1000 dilution) followed by three washes of TBST. Then, the blots were incubated with horseradish peroxidase- (HRP-) conjugated secondary antibody (1 : 2000 dilution) for 2 h at room temperature followed by three washes using TBST. Finally, the membranes were visualized using a Clarity Western Enhanced Chemiluminescence kit (Bio-Rad Laboratories Inc., CA, USA) and an automatic chemiluminescence image analysis system (Tanon Science & Technology Co., Ltd., Shanghai, China).

### 2.9. Quantitative Real-Time PCR Analysis

Total RNA was extracted from liver tissues using TRIzol Reagent (Invitrogen, Life Technologies, CA, USA) according to the manufacturer's protocols. The purity of RNA was measured using Thermo Scientific NanoDrop 2000 (MA, USA). Next, the RNA was reverse transcribed to cDNA using a reverse transcriptase kit (TaKaRa Biotech, Kyoto, Japan). The quantitative real-time PCR (qRT-PCR) analysis was performed using a ChamQ SYBR qPCR Master Mix (Vazyme Biotech Co., Ltd., Nanjing, China) with the CFX Manager software (Bio-Rad Laboratories Inc.). GAPDH was analyzed in each sample to normalize expression. The primers used in this study are listed in Table 1. The relative expression was analyzed by the 2^−ΔΔCt^ method.

### 2.10. Statistical Analysis

All the experimental data were represented as means ± standard error of the mean (SEM). One-way analysis of variance followed by the multiple comparisons of least significant difference test was used for all the data analysis. The analysis was performed using the Statistical Product and Service Solutions software 20.0 (SPSS Inc., NY, USA). A *P*value < 0.05 or <0.01 was considered as statistically significant.

## 3. Results

### 3.1. Determination of Carbohydrate Content in DOP

Dry polysaccharides (5.15 g) were obtained from *D. officinale* (100 g). The yield of DOP was 21.07%. The carbohydrate content was determined based on the established linear curve of D-glucose [*Y* = 0.0054*X* − 0.0354 (*R*^2^ = 0.9992)]. The content of total carbohydrate in DOP was 29.52 ± 0.99%.

### 3.2. Determination of MW of DOP Using HPGPC

The MW of DOP was based on the calibration curve of dextran standards [log MW = −0.4891 V + 13.975 (*R*^2^ = 0.9981)]. The MW of DOP was found to be 8551 Da.

### 3.3. DOP Alleviated APAP-Induced Liver Injury

**Figure 1:**
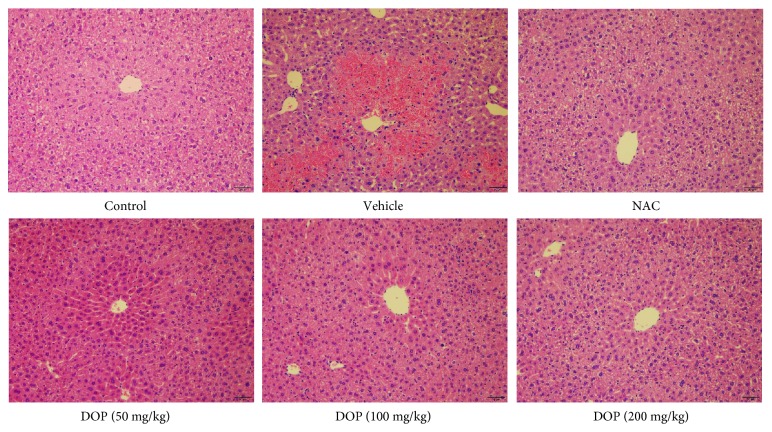
Results of histopathological examination of the mouse liver sections (H&E staining, magnification 200x). Scale bar indicates 50 *μ*m.

The mice were orally given DOP in advance to investigate the effects of DOP on APAP-induced hepatotoxicity. The histopathologic features of H&E-stained liver sections are shown in Figure 1. Clear hepatic lobules and nuclei and no inflammation infiltration were observed in the control group. However, massive hepatic necroses, focal intrahepatic hemorrhage, and inflammation were noted around the central venous lesions in APAP-treated mice. Meaningfully, DOP treatment significantly ameliorated APAP-induced hepatotoxicity.

### 3.4. DOP Alleviated APAP-Induced Liver Enzyme Dysfunction

**Figure 2:**
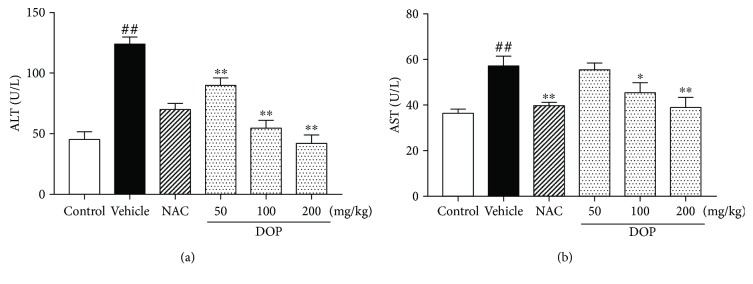
Effect of polysaccharides isolated from *Dendrobium officinale* (DOP) on the serum levels of alanine transaminase (ALT) (a) and aspartate aminotransferase (AST) (b) in mice. Data are presented as mean ± SEM (*n* = 6). ^##^*P* < 0.01 versus the control group. ^∗^*P* < 0.05 and ^∗∗^*P* < 0.01 versus the vehicle group.

As shown in Figure 2, the levels of ALT and AST significantly increased in the vehicle group following APAP challenge (*P* < 0.01) compared with the control group. However, DOP (100 and 200 mg/kg) treatment significantly reversed these changes (*P* < 0.01, *P* < 0.05). These data indicated that DOP alleviated APAP-induced liver enzyme dysfunction.

### 3.5. DOP Suppressed APAP-Induced Oxidative Stress

**Figure 3:**
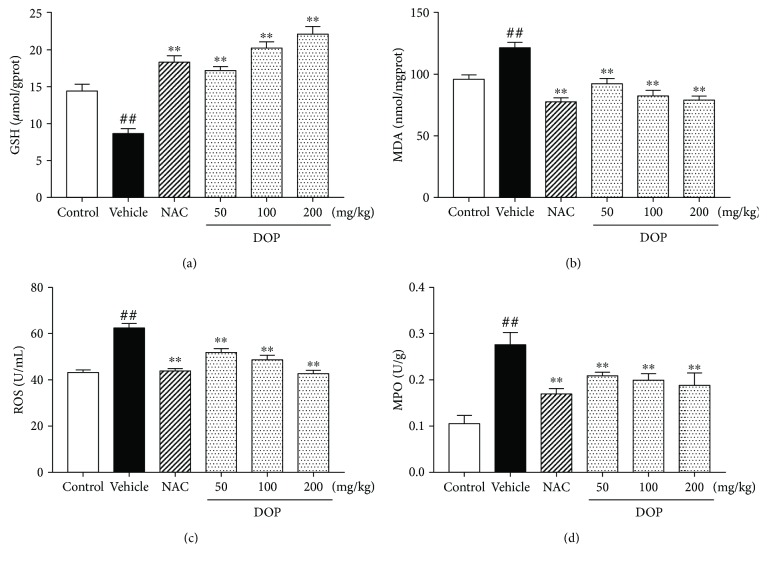
Effect of polysaccharides isolated from *Dendrobium officinale* (DOP) on glutathione (a), malondialdehyde (b), and reactive oxygen species (c) levels and myeloperoxidase activity (d). Data are presented as mean ± SEM (*n* = 6). ^##^*P* < 0.01 versus the control group. ^∗∗^*P* < 0.01 versus the vehicle group.

Previous studies showed that oxidative stress was closely associated with liver injury. As shown in Figure 3(a), the GSH level in the vehicle group was significantly lower than that in the control group (*P* < 0.01). Beside, NAC treatment significantly increased the GSH level compared with that in the vehicle group (*P* < 0.01). DOP treatment also significantly increased the GSH level in the liver compared with that in the vehicle control (*P* < 0.01). The GSH levels in the moderate and high doses of DOP groups were much higher than those in the NAC group.

As shown in Figures 3(b)–3(d), APAP stimulation significantly increased the levels of MDA and ROS and MPO activity in the liver compared with the control group (all, *P* < 0.01). Compared with the vehicle group, the NAC group significantly reduced the levels of MDA and ROS and MPO activity (all, *P* < 0.01). DOP treatment also reduced the productions of MDA and ROS and the activity of MPO (all, *P* < 0.05). These results suggested that DOP strongly suppressed APAP-induced hepatic oxidative stress.

### 3.6. DOP Increased the Antioxidative Capacity against APAP-Induced Oxidative Stress

**Figure 4:**
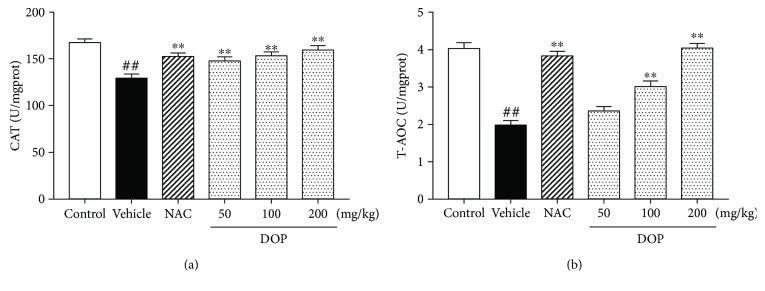
Effect of polysaccharides isolated from *Dendrobium officinale* (DOP) on catalase (a) and total antioxidant capacity (b). Data are presented as mean ± SEM (*n* = 6). ^##^*P* < 0.01 versus the control group. ^∗∗^*P* < 0.01 versus the vehicle group.

As shown in Figures 4(a) and 4(b), the CAT and T-AOC levels in the vehicle group (APAP) were significantly lower than those in the control group (all, *P* < 0.01), whereas NAC upregulated the levels of CAT and T-AOC (all, *P* < 0.01). Interestingly, three doses of DOP increased the CAT level (all, *P* < 0.01), and DOP (100 and 200 mg/kg) increased the T-AOC level (*p* < 0.01) compared with the vehicle group. However, DOP (50 mg/kg) had no effect on liver T-AOC level. The data also indicated that DOP had a powerful antioxidative capacity against APAP-induced oxidative stress.

### 3.7. Effect of DOP on Nrf2 Nuclear Translocation

**Figure 5:**
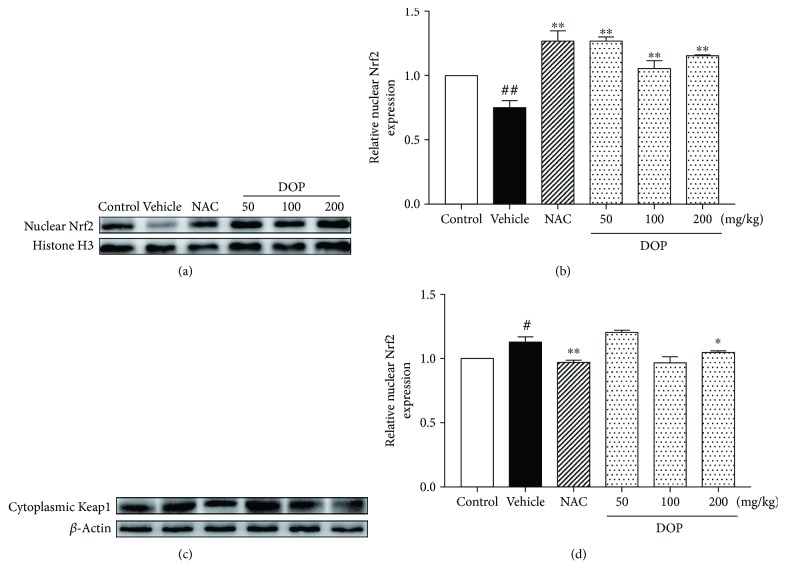
(a–c) Western blot analysis was used to measure nuclear Nrf2 and cytoplasmic Keap1 protein expression. Specific band intensities were quantified and normalized to histone H3 and *β*-actin, respectively (b–d). Data are presented as mean ± SEM (*n* = 3). ^#^*P* < 0.05 and ^##^*P* < 0.01 versus the control group. ^∗^*P* < 0.05 and ^∗∗^*P* < 0.01 versus the vehicle group.

Nrf2 nuclear translocation and cytoplasmic Keap1 were determined using Western blot analysis to estimate whether DOP affected the Nrf2−Keap1 signaling pathway. As shown in Figures 5(a) and 5(b), APAP stimulation caused a decrease in Nrf2 level in nuclei in the mice compared with the control group (75.1%, *P* < 0.01). However, DOP significantly promoted the Nrf2 nuclear translocation compared with the APAP group. The change in level Nrf2 with DOP treatment was as follows: 50 mg/kg, 0.27-fold increase (*P* < 0.01); 100 mg/kg, 0.06-fold increase (*P* < 0.01); and 200 mg/kg, 0.16-fold increase (*P* < 0.01). Besides, APAP alone also caused an increase in cytoplasmic Keap1 (112%, *P* < 0.05). Nevertheless, the expression level of cytoplasmic Keap1 was significantly reduced with 200 mg/kg DOP treatment compared with the APAP group (*P* < 0.05). These results demonstrated that DOP enhanced the nuclear translocation of Nrf2.

### 3.8. Effect of DOP on the Expression of Nrf2 Target Gene in the Liver

**Figure 6:**
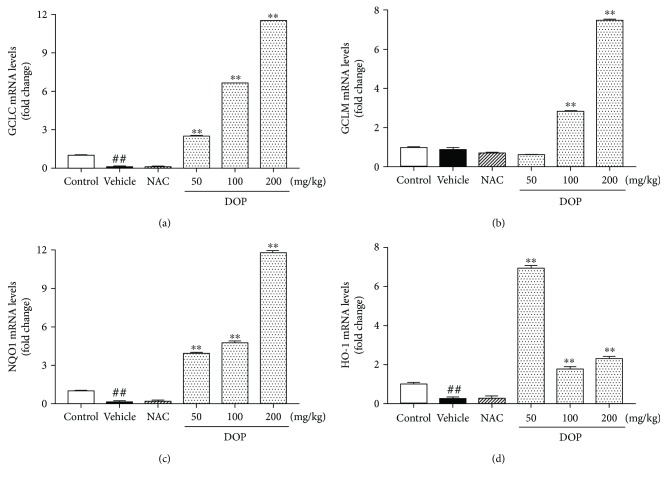
Effects of polysaccharides isolated from *Dendrobium officinale* (DOP) on the mRNA expression levels of GCLC (a), GCLM (b), NQO1 (c), and HO-1 (d). Total RNA from liver tissues was extracted and reverse transcribed into cDNA prior to quantitative real-time PCR analysis to detect mRNA expression of GCLC, GCLM, NQO1, and HO-1. Data are presented as mean ± SEM (*n* = 3). ^##^*P* < 0.01 versus the control group. ^∗∗^*P* < 0.01 versus the vehicle group.

Four indexes, including GCLC, GCLM, HO-1, and NQO1, in the liver samples were measured using qRT-PCR to investigate the effect of DOP on Nrf2 target genes. As shown in Figure 6, APAP stimulation resulted in decreases in the mRNA expression levels of GCLC, GCLM, HO-1, and NQO1. Nevertheless, DOP treatment significantly upregulated the mRNA expression levels of GCLC, GCLM, HO-1, and NQO1 compared with the APAP group. Among three doses of DOP, 200 mg/kg DOP displayed the strongest promotion in the mRNA expression levels of GCLC (1152%), GCLM (747%), NQO1 (1180%), and HO-1 (229%) (all, *P* < 0.01).

## 4. Discussion

The present study investigated the protective effects of DOP against APAP overdose-induced hepatotoxicity. The results indicated that DOP exerted the hepatoprotective effect by alleviating the oxidative stress through the Nrf2–Keap1 signaling pathway.

First, the dose of APAP was optimized. The study found that 230 mg/kg APAP was able to cause significant and stable hepatotoxicity in the preliminary experiment. Hence, 230 mg/kg APAP was used to induce liver injury in the following study. ALT and AST, two indicators of liver function, reflect the degree of liver injury [29]. Obviously, APAP administration significantly increased the levels of ALT and AST (shown in Figure 2), whereas DOP treatment significantly inhibited the activities of ALT and AST in serum. Furthermore, histopathological analysis of liver tissues also revealed that DOP treatment reduced hepatic necroses and focal intrahepatic hemorrhage, besides arranging hepatic lobules in neat rows. The aforementioned results demonstrated that DOP exerted protective effects against APAP-induced liver injury.

An increasing body of evidence demonstrated an important role of oxidative stress in the liver injury induced by APAP [30–32]. Primarily, using cytochrome P450 enzymes, APAP-induced hepatotoxicity is ascribed to the formation of *N*-acetyl-*p*-benzoquinone imine (NAPQI), the toxic metabolite that causes hepatic glutathione (GSH) depletion and oxidative stress [33, 34]. Afterward, residual NAPQI combines with mitochondrial proteins and causes mitochondrial dysfunction and ROS overproduction, finally resulting in DNA fragmentation and liver injury [35]. In addition, lipid peroxidation (LPO) has been a frequently invoked mechanism in ROS-induced cell death and liver injury [36]. Importantly, the massive LPO caused massive liver injury and acute liver failure within 4 h after APAP overdose [37]. Besides, GSH, ROS, MDA, CAT, and T-AOC levels are considered as the indicators of liver function [27, 38]. Lipid peroxidation was assessed by estimation of MDA in the liver tissues [39].

In the present study, APAP challenge significantly decreased the levels of GSH, CAT, and T-AOC in the mice compared with the control. Meanwhile, the production of ROS and MDA in the mice treated with APAP increased significantly. However, DOP treatment significantly upregulated the GSH, CAT, and T-AOC levels and downregulated the production of ROS and MDA, suggesting that DOP alleviated the hepatic oxidative stress induced by APAP. MPO activity is another marker of oxidative stress and inflammation [40]. The activity of MPO in the mice increased significantly after the APAP insult, leading to serious liver inflammation. However, DOP treatment significantly inhibited the activity of MPO. Combining these results, it was concluded that DOP treatment attenuated APAP-induced hepatic oxidative stress.

Next, the present study investigated the underlying mechanism of DOP against APAP-induced liver injury. Nrf2, a main regulator of the antioxidant defense system, mediates the antioxidant response element (ARE) [41]. On the contrary, Keap1 is a negative regulator of Nrf2 that combines with Cullin 3-based E3 ubiquitin ligase and results in the degradation of Nrf2 [42]. When activated, Nrf2 translocates from the cytoplasm to the nucleus and regulates the expression of intracellular detoxifying and antioxidant genes, such as GCLC, GCLM, HO-1, and NQO1 [43, 44]. Glutamate–cysteine ligase, consisting of GCLC and GCLM subunits, was reported to facilitate the synthesis of GSH [45]. NQO1 could reduce the NAPQI production and subsequently alleviate mitochondrial dysfunction caused by APAP [46]. HO-1 was demonstrated to promote the cleavage of heme to accelerate biliverdin formation and reduce intracellular ROS production [47]. Furthermore, the Nrf2–ARE signaling pathway was demonstrated to be involved in the hepatotoxicity induced by APAP [48]. Previous studies showed increased protein and mRNA expression levels of Nrf2 after effective drug treatment against APAP [41, 49, 50]. Importantly, the present study found that DOP treatment could increase the mRNA expression level of Nrf2 and also upregulate the expression of GCLC, GCLM, NQO1, and HO-1 compared with the APAP group. Moreover, DOP treatment could catalyze Nrf2 nuclear translocation and reduce the expression of Keap1, suggesting that DOP might exert a protective effect against APAP-induced hepatotoxicity via activating the Nrf2–Keap1 pathway.

## 5. Conclusions

This novel study demonstrated that DOP treatment exerted potentially hepatoprotective effects against APAP-induced liver injury. Further investigation of the underlying mechanisms indicated that DOP exerted the hepatoprotective effect by suppressing the oxidative stress and activating the Nrf2–Keap1 signaling pathway. Collectively, DOP may be developed into a potential hepatoprotective drug, diet therapy, or functional food against APAP overdose-induced liver injury.

## Figures and Tables

**Table 1:** Primers used in this study.

Primer name	Primer sequence (5′-3′)	Product size
GCLC forward	GCACATCTACCACGCAGTCAAGG	273
GCLC reverse	GCCTCCACAGTGTTGAACTCAGAC	273
GCLM forward	GCTTCGCCTCCGATTGAAGATGG	380
GCLM reverse	ACGATGACCGAGTACCTCAGCAG	380
NQO1 forward	TCACAGGTGAGCTGAAGGACTCG	367
NQO1 reverse	CGCAGGATGCCACTCTGAATCG	367
HO-1 forward	TCTGGTATGGGCCTCACTGG	350
HO-1 reverse	AATGTTGAGCAGGAAGGCGG	350
GAPDH forward	AATGGTGAAGGTCGGTGTGAACG	235
GAPDH reverse	TCGCTCCTGGAAGATGGTGATGG	235

## Data Availability

The data used to support the findings of this study are available from the corresponding author upon request.
